# Role of Cytokeratin 20 as a Predictive and Prognostic Marker in Urothelial Neoplasms

**DOI:** 10.7759/cureus.31607

**Published:** 2022-11-17

**Authors:** Hristo Popov, George S Stoyanov, Peter Ghenev

**Affiliations:** 1 General and Clinical Pathology, Forensic Medicine and Deontology, Medical University of Varna, Varna, BGR; 2 General and Clinical Pathology, St. Marina University Hospital, Varna, BGR

**Keywords:** recurrence, progression, tumor grade, tumor staging, cytokeratin 20, bladder, non-invasive urothelial carcinoma, urothelial carcinoma

## Abstract

Introduction

Several clinical peculiarities mark urothelial carcinomas and their biological behavior. Key in these are its relatively indolent course before manifestation and its high recurrence rate. So far, no biomarker has been identified as a predictor for these factors. The current study aims to evaluate the role of cytokeratin 20 (CK20) in non-invasive urothelial carcinomas (pTa and pT1) of the urinary bladder and its diagnostic and predictive role in tumor staging and recurrence.

Materials and methods

The study utilizes a retrospective, non-clinical approach via immunohistochemical marking of the paraffin-embedded tumor tissues for the initial diagnosis. Expression patterns were compared with tumor grade and stage, as well as the incidence of recurrence within a five-year follow-up period.

Results

A strong statistical correlation was established between expression and tumor grade, with high-grade tumors showing weak to moderate expression of CK20 while low-grade tumors showed an intensive expression pattern. No correlation was noted between the expression pattern, patient age and gender, tumor stage, and the likelihood of local recurrence.

Conclusion

While CK 20 is a reliable diagnostic marker when used together with other markers, its expression pattern in our study correlated only with bladder urothelial carcinoma grade.

## Introduction

The clinical behavior of urothelial carcinoma, compared to other malignant tumors, is marked by a myriad of peculiarities, such as a prolonged period before clinical manifestation, superficial and/or rapid invasive growth, high rate of recurrence, and a five-year survival rate of around 70% for invasive versus more than 90% for non-invasive forms [1-4].

All this necessitates the search for new approaches in determining the biological behavior of urothelial malignancies and establishing new prognostic and predictive markers for these tumors. As reported by some studies, one marker showing promise in this area is the expression of Cytokeratin 20 (CK20) by the tumor cells [5,6].

CK 20 belongs to the family of cytokeratins - cytoplasmic low- and high-molecular-weight intermediate filaments. They are located in the cytoplasm of multiple types of epithelial cells [5,7]. Regarding urothelium, CK20 is typically expressed in the superficial (umbrella) cells and some intermediate cells in the bladder mucosa. In cases of reactive atypia, CK20 shows expression only in the superficial layer of cells. In contrast, CK20 is expressed diffusely throughout the mucosal layer in carcinoma in situ in around 70%-80% of cases [5]. There are also conflicting reports on the potential role of CK20 as a marker used for the grading and staging of urothelial carcinoma in low-grade (G1-2) and high-grade (G2-G3) [6].

The present study aims to evaluate the expression of CK20 urothelial neoplasms and compare expression location and intensity between different grades, recurrence, progression, and survival groups.

## Materials and methods

Study design

A retrospective and non-clinical approach was selected for the means of the study.

Patient selection

The study was carried out on routine formalin-fixed paraffin-embedded endoscopic biopsy specimens stored in the histo-bank of the Department of General and Clinical Pathology of the St. Marina University Hospital, Varna, Bulgaria and which were used for the initial histopathological diagnosis. Inclusion criteria were the primary diagnosis of urothelial carcinoma of the urinary bladder in our institution in stages pTa and pT1. Exclusion criteria were the placement of the initial diagnosis in another healthcare institution for patients monitored at our hospital and the presence of a higher stage malignancy, second histogenetically different malignancy, or non-urinary bladder urothelial carcinoma. A total of 163 patients fit the inclusion criteria without contradicting the excluded ones and were split into two groups based on their stage: pTa or pT1.

All patients were initially diagnosed between 2007 and 2011 and monitored for five-calendar years. Upon initial diagnosis, all patients were administered an intravesicular Bacillus Calmette-Guérin vaccine (Merck & Company, US). Patient follow-up included cystoscopy every six months or upon the development of clinical complaints, mainly hematuria, with transurethral resection if recurrence was noted.

Histological slide preparation and interpretation

An indirect immunoperoxidase method was utilized for immunohistochemical (IHC) analysis using a primary CK20 antibody (catalog number IR777; Dako, Aligent technologies, Santa Clara, California, US) and the DAKO mini KIT high Ph visualizing system (catalog number K8024; Aligent technologies, Santa Clara, California, US), an anti-polyvalent 3,3'-Diaminobenzidine detection system. All slides were compared with the initial H&E stained slides used for the initial diagnosis, with IHC interpretation performed on an Olympus BX50 light microscope (Olympus Optical Co, Ltd., Shinjuku, Tokyo, Japan). The H&E slides were reevaluated for the histological type of tumor as per the 2022 World Health Organization and International Society of Urological Pathology criteria for urothelial malignancies and staged based on the 2018 American Joint Committee on Cancer guidelines.

The evaluation of the IHC reaction was performed by evaluating 10 fields at 400x magnification for each case, with the reaction intensity and percentage of positive cells being assessed using a histo-score (H-score) using the following formula:

H-score = (0 x % of I0) + (1 x % of I1+) + (2 x % of I2+) + (3 x % of I3+)

The intensity (I) of the reaction was determined for each cell in the different fields ranging from negative (0) to weak (1), moderate (2) and intense (3). Calculated in this way, the H-score varies from 0 to 300. The final score was dichotomously divided according to the median (determination of cut-off value); all cases above the median were reported as high expression levels and those below the median as low.

Statistical analysis

The statistical analysis was carried out using the MS Excel 2016 (Redmond, USA) programming product and the IBM Corp. Released 2017. IBM SPSS Statistics for Windows, Version 25.0. Armonk, NY: IBM Corp. for Windows. Descriptive analysis for determining statistical parameters included mean [μ(X)], standard deviation (SD), minimum (min), and maximum value (max).

Cross-tabulation and chi-square analysis were performed for significant differences in the frequency performance of category values. Statistical significance in the chi-square test was considered at p ≤ 0. 05. Correlation analysis was conducted to assess the relationship between the indicators examined and to establish the strength of the interaction. The degree of association between variables was defined as significant at r > 0.5 < r = 0.7, large at 0.7 < r = 0.9, and extremely large at r > 0.9 at p ≤ 0.05. Kaplan-Meier test was conducted for the survival of patients with urological carcinoma according to the influence of low and high expression of antibodies; and the clinical-morphological indicators studied. A student's t-test was conducted to compare quantitative and qualitative indicators and examine their differences. The resulting data was presented as an arithmetic mean; and SD for the groups studied. Receiver operating characteristic (ROC) curve analysis was done to determine the role of accuracy and specificity of the predictability of certain indicators. The value of the area under the curve was between 0.5 and 1.0. Complete separation of values by an indicator was done with a score above 0.75 or 75%.

In all analyses, a permissible level of significance of p < 0.05 at a 95% confidence interval (CI) was assumed. The graphical and tabular presentation of statistics was performed in Microsoft Office 2016 (One Microsoft Way, Redmond, Washington, US).

Ethical approval

All procedures carried out in the study fully adhered to the ethical standards of the Helsinki declaration of 1975 and its seventh revision in 2013, as well as the ethical standards of the Bulgarian Ministry of Healthcare and the Bulgarian Medical Association. The study received ethical approval, protocol number 54/19.05.2016, issued by the Medical University - Varna Committee on Scientific Ethics.

## Results

Recurrence frequency

The tumors were divided into non-recurrent and recurrent groups based on the disease course over the five-year follow-up period. The first group consisted of 95 cases, and the second one of 68 cases. Based on this data, our cohort's five-year local recurrence rate of pTa and pT1 urothelial bladder carcinoma is 41.72% and was not statistically associated (p>0.05) with tumor stage.

CK 20 expression

**Figure 1:**
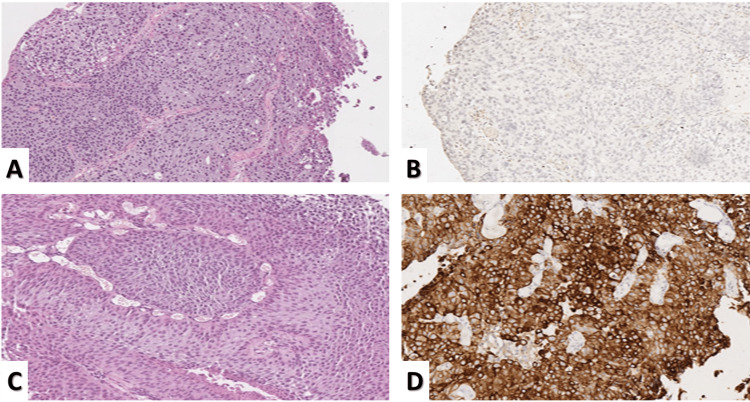
CK20 expression in urothelial carcinoma A: high-grade urothelial carcinoma, H&E stain, original magnification 40x; B: same tumor from A with CK20 expression, CK20 IHC reaction, original magnification 40x; C: low-grade urothelial carcinoma, H&E stain, original magnification 40x; D: same tumor from C with a diffuse, intense reaction with CK20, CK20 IHC reaction, original magnification 100x. CK20: cytokeratin 20; H&E: hematoxylin and eosin; IHC: immunohistochemistry

CK20 expression was noted to be highly heterogenous within tumor samples, with some tumors even lacking expression (Figure 1).

**Figure 2:**
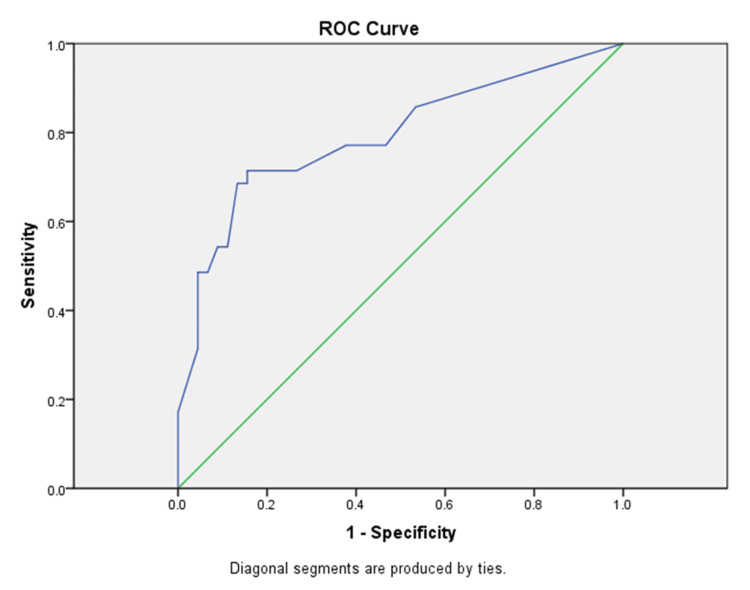
ROC Surve analysis with a cut-off value of 135 for CK20 and local recurrence (AUC = 0.792, 95% CI: 0.688-0.896, p <0.001) ROC curve: receiver operating characteristic curve; CK20: cytokeratin 20; AUC: area under the curve; CI: confidence interval

Expression levels differed between the two groups of urothelial carcinomas: low (G1-2) and high-grade tumors (G2-3), with high-grade tumors showing less intensive cytoplasmic immunohistochemical reactions with CK20 than low-grade ones. ROC curve analysis showed that a cut-off value of 135 was optimal for distinguishing between the two groups, with a sensitivity of 72.7% and specificity of 78.7% (AUC = 0.792, 95% CI: 0.688-0.896, p <0.001), (Figure 2).

Further statistical analysis showed no significance between CK20 expression and age, gender, tumor stage, and occurrence of local recurrence when p<0.05 was considered statistically significant.

## Discussion

While CK20 is by no means a new biomarker, it has been reported to be of diagnostic use for urothelial malignancies [8-10]. It is, however, essential to remember that CK20 is not a marker for urothelial differentiation, as it is also expressed in a myriad of other malignant conditions ranging from colorectal adenocarcinoma to ovarian and Merkel cell malignancies [11].

The results presented here confirm the multidimensional role of CK20 in the different aspects of early bladder carcinoma behavior. From one side, the positive expression of CK20 cannot be used as a marker of histogenesis, but it is statically significant (ROC curve analysis) for evaluating the tumor grade (low grade versus high grade). The CK20 expression pattern in high-grade urothelial carcinomas shows low to intermediate expression (almost absent), in contrast to low-grade urothelial carcinomas, which show diffuse intensive expression [7]. Authors such as Desai S et al., evaluating the expression of CK20 in 42 low-grade and 62 high-grade urothelial carcinomas, found that it was associated with high-grade carcinomas and tumor stage [12]. In contrast, we found no relationship between CK20 expression and tumor stage and the results concerning the tumor grading.

Furthermore, Jiang et al. show that metastatic urothelial carcinoma retains its CK20 expression, unlike other markers and malignant sites, which lose some of their biomarker expressions [9]. As demonstrated by Akhtar et al., CK20 and other biomarkers can also be used as a prognostic marker, as retained CK20 and loss of other cytokeratins are associated with more aggressive biological behavior [10]. As seen by our results and the studies mentioned, CK20 is a more reliable marker for tumor grade but can also be used in the context of other markers to determine the urothelial origin of a metastatic tumor and its biological potential.

The lack of correlation with patient age and gender, progression-free survival, and tumor stage, as seen by our results, should not discourage the use of CK20 in routine diagnostic histopathology. The cut-off value is statistically significant for tumor grading in non-invasive forms, making CK20 a promising pathological biomarker. Future studies should also be encouraged to evaluate its role in grading invasive tumor (T2 and beyond) forms and establishing its predictive role for stage and risk of recurrence and metastasis.

Study limitations

The main limitations of our work are the relatively small number of patients included and its retrospective nature. While the follow-up period of the patients is over a five-year interval, the number of patients excluded due to contradicting the exclusion criteria is high, and in a larger cohort, the statistical significance of the established markers may differ. Furthermore, as we focus only on the urinary bladder, the CK20 correlations established may be valid only for that location, as tumors located in the ureters and renal pelvis, which already differ in their clinical progression, despite being in the same histogenic group, may show different correlations.

A further limitation of our study is the use of a single antibody to evaluate the expression pattern (catalog number IR777; Dako, Aligent technologies, Santa Clara, California, US). The difference in expression pattern to other studies may be due to the loss of the binding site of the CK20 protein and not due to the actual loss of the intracellular protein. As different manufacturers produce antibodies that bind to a different site of the protein chain, the loss of expression may be due to minor changes to the chain, and hence future studies should also be encouraged to evaluate the tumor proteomics and/or coding nucleic acid within urothelial neoplasms.

## Conclusions

In summary, the results obtained in the study of 163 patients with urothelial carcinoma in stage pTa and pT1 found data confirming, on the one hand, those in the literature, and on the other - pointed to some new circumstances related to the progression of urothelial cancer - high-grade tumors have a weaker expression pattern, which does not correlate with tumor progression or recurrence.

Although the suggested mechanisms reveal different aspects of urothelial carcinoma biology and need further clarification, the results presented here provide statistically significant evidence that CK20 expression intensity in the primary biopsy is a reliable factor for tumor grading.
